# Natural Death

**Published:** 1878-11

**Authors:** 


### NATURAL DEATH,

Is to die sweetly without a sob, a struggle, or a sigh. It is the result of a long life of uninterrupted health, of a long life of "*temperance in all things*," and such a death should be one of the ends and aims of every human being, so that we may not only live long, but in that long life be able to do much for man, and much for God.

The love of life is a universal instinct; life is a duty, its peril or neglect a crime; to be anxious to die is the feeling of a coward or a loafer. We are placed on earth for a purpose, that purpose can be none other than to give us an opportunity of doing good to ourselves and to others, and to be anxious to be "*off duty*," sooner than God wills, is no indication of true piety. The good man has one ruling, ever present desire, and that is to live as long on the earth as his Maker pleases, and while living to do the utmost he can to benefit and bless mankind; and to accomplish a long and active, and useful life, the study how to preserve and promote a high degree of bodily health is indispensable. And it seems to have been ordained by a Providence both kind and wise, as a reward of a temperate life, that such a life should be largely extended, and that its decline should be as calm as a summer's evening, as gentle as the babe sleeps itself away on its mother's bosom.

These sentiments were advocated in one of the first numbers of the Journal in the way of argument to induce its readers to live temperately in all things, in order by length of life to secure the largest leisure for doing the greatest amount of good, and as a beautiful illustration of the fact, how easily the very old may die, I give here a letter written by the Rev. Dr. Green of Tennessee, to the able Editor of the Nashville Medical Journal.

I promised you that I would furnish you with some of the facts connected with the last days of Aunt Phillis, an old negro woman of mine, who died last fall. Aunt Phillis was at the time of her death, at the lowest estimate, 111 years old, and the probability is that she was several years older.

For fifty years she has enjoyed uninterrupted health, and, as far as I have been able to learn, she was never sick in her life, except at the birth of her children. For thirty years of her life, and down to within three years of her death, she did not seem to undergo the slightest change in her appearance—time exercising but little power over her. The first sign of decay was that of sight, which took place about three years before her death; up to that time she was in the full enjoyment of all her senses; and at one hundred and four years would have married an old negro man of seventy-five if I had not objected.

Her sight failed not in the usual way, but she became near-sighted, not being able to see objects at a distance. Soon after this her hearing declined, but up to the time of her death she could hear better than old persons generally do. The first indication of mental failure was that of locality, she not being able to find her way to a neighbor's house; yet her memory seemed perfect in all other respects. She recollected her friends and old acquaintances, but could not find her way to their houses.

I at first supposed this was owing to defective sight, but on examination found it was in the mind. Still her locomotion was good; she had the full use of herself, and could walk strong and quick like a young person, and held herself up so straight that, when walking from me, I often took her for some of the younger servants about the premises. The next, and to me the most singular sign of decline was, that she lost the art of walking—not that she had not strength enough to walk, but forgot how to walk.

The children would lead her forth and interest her for a while, and she would get the idea, which seemed to delight her very much, and she would walk about the yard and porches until some person would tell her she had walked enough—but she would no sooner take her seat, and sit for a few moments, before all idea of walking would be gone, and she would have to be taught over again.

At length she became unwilling to try to walk unless she had hold of something; take her by the arm and she would walk, and walk well, but just as soon as you would let her go she would stop, and if no further aid was afforded her she would get down and crawl like a child; and at length became so fearful that she refused to walk altogether, and continued to sit up during the day, but had to be put to bed and taken up like a child. After a while she became unwilling to get up altogether, and continued to lie until she died.

All this time she seemed to be in good health, took her regular meals, and her stomach and bowels were uniformly in good condition. I often examined her the best I could, and she had no pains, no sickness, no aches of any kind, and from her own account, and from all that I was able to learn, she was in good health and all the while in fine spirits. The intellect and the mind seemed to be perfectly good, only that she did not seem to know where she was all the time.

At length one of the children said to me that Aunt Phillis was getting cold, and on examining her I found it even so; the extremities were cold—still she took her regular meals; and did not complain of anything; and the only change that I recollect of was that she slept a little more than usual. The coldness increased for two days, when she became as cold almost as a dead person. Her breathing began at length to shorten, and grew shorter and shorter till she ceased to breathe.

Death closed in upon her like going into a soft, sweet sleep, and for two minutes it was difficult to tell whether she was breathing or not. There was no contortion, no struggle, no twisting of the muscles, but after death she might have still been taken, on a slight examination, to have been in a deep sleep. So passed away Phillis—the only natural death I ever witnessed.

